# ProAna Worlds: Affectivity and Echo Chambers Online

**DOI:** 10.1007/s11245-021-09785-8

**Published:** 2021-12-12

**Authors:** Lucy Osler, Joel Krueger

**Affiliations:** 1 Center for Subjectivity Research, Department of Communication, University of Copenhagen, Karen Blixens Plads 8, 2300 Copenhagen S, Denmark; 2 Department of Sociology, Philosophy, and Anthropology, University of Exeter, Amory, Rennes Drive, Exeter, EX4 4RJ UK

**Keywords:** Anorexia nervosa, ProAna communities, Online communities, Echo chambers, Affectivity, Nguyen, Lugones

## Abstract

Anorexia Nervosa (AN) is an eating disorder characterised by self-starvation. Accounts of AN typically frame the disorder in individualistic terms: e.g., genetic predisposition, perceptual disturbances of body size and shape, experiential bodily disturbances. Without disputing the role these factors may play in *developing* AN, we instead draw attention to the way disordered eating practices in AN are *actively supported* by others. Specifically, we consider how Pro-Anorexia (ProAna) websites—which provide support and solidarity, tips, motivational content, a sense of community, and understanding to individuals with AN—help drive and maintain AN practices. We use C. Thi Nguyen’s work on epistemic “echo chambers”, along with Maria Lugones’ work on “worlds” and “ease”, to explore the dynamics of these processes. Adopting this broader temporal and intersubjective perspective, we argue, not only helps to further illuminate the experiential character of AN but also has important clinical and therapeutic significance.

## Introduction

Anorexia Nervosa (AN) is an eating disorder characterised by self-starvation. AN has received significant medical and academic attention and yet is still poorly understood. The incidence of AN is on the rise (Galmiche 2019), it is thought to have the highest mortality rate of any psychiatric disorder (Arcelus et al. 2011), and the relapse rate for those with the disorder remains high (Carter et al. 2004). There is now additional concern that lockdowns due to Covid-19 also aggravate disordered eating (Shah et al. 2020; Touyz et al. 2020).

Accounts of AN typically focus on the disorder in *individualistic* terms: e.g., how one is genetically or psychologically predisposed to having an eating disorder (e.g. Bulik et al. 2007), how one (mis)perceives one’s own body size and shape (e.g. Cash and Pruzinsky 2004), how one experiences one’s body (e.g. Fuchs 2021; Legrand 2010; Osler 2021a, b; Rodemeyer 2021), and so on. Where other people come into the picture is typically at the level of causation. For instance, feminist models emphasise the role of society in promoting thinness as a feminine ideal (Bowden 2012; Bordo 1992; Orbach 1978), while others focus on the role that controlling or hostile family relationships play in the emergence of eating disorders (e.g. Byely et al. 2000; Legrand and Briend 2015; Paxton and Eisenberg 2006). Without wanting to dispute the role these factors play, we draw attention not to the way disordered eating practices^1^ in AN *arise* but to a way in which they are *supported* and *sustained* by others.^2^ Adopting this broader temporal and intersubjective perspective, we argue, not only helps to illuminate the experiential character of AN but has potential clinical and therapeutic significance, too.

We take as our focus the role that the Internet plays in providing a variety of platforms, tools, and communities that can help individuals sustain disordered eating practices. Specifically, we consider how Pro-Anorexia (ProAna) websites—sites that provide interpersonal support and solidarity, tips, motivational content (such as so-called “thinspiration”), a sense of community, and understanding to individuals with AN—support, shape, and maintain AN. In the media and the medical community, ProAna sites are typically treated as dangerous informational resources that provide “how-to” guides for AN. We argue that this picture is too reductive and static to do justice to the role that such sites play in the lives of those with AN. More specifically, this picture fails to: capture the complexity of such sites; give a satisfactory account of why members frequent these sites after they have obtained the information required; and, finally, explain why they may be so difficult for members to leave. In this paper, we provide a fine-grained and dynamic analysis of these ProAna sites. We argue that these online resources create *ProAna worlds* with epistemic and affective significance. Importantly, both the epistemic and affective dimensions of these ProAna worlds, we argue, construct barriers to an individual leaving.

In Sect. 1, we introduce the ProAna websites and cross-platform activities that many with AN frequent. We note that the primary medical response to these ProAna sites is that they should be regarded as dangerous sources of information promoting disordered eating practices. In Sect. 2, we use C. Thi Nguyen’s (2020) work on echo chambers to highlight that ProAna sites are interactive epistemic communities that generate certainty about ProAna beliefs and commitments, while simultaneously excluding and discrediting voices outside the community. Thus, ProAna communities work as “echo chambers” that sediment and scaffold AN beliefs and practices, and generate a trust barrier that can work to hinder therapeutic intervention. However, while this move goes some way to enriching our understanding of ProAna communities, we argue further that these communities aren’t *just* epistemic echo chambers. Individuals with AN gravitate towards ProAna sites and stay within these communities not just for epistemic reasons but also because these sites have a certain *affective allure*. They offer communities in which individuals with AN feel a sense of comfort, belonging, and identity. These social and affective dimensions make these communities attractive and “sticky” spaces. In Sect. 3, we integrate Nguyen’s concept of echo chamber with Maria Lugones’ (1987, 2003) framework of “worlds”. With these concepts in hand, we suggest we can capture how these ProAna resources create *intersubjective affective epistemic worlds* and explore the therapeutic implications of our account.

## Medicalising and Moralising ProAna Sites

AN is classified as an eating disorder by the American Psychiatric Association (APA). It is characterised by self-starvation that leads to dramatic weight loss.^3^ Within AN literature, increased attention has been given to the rise and proliferation of online spaces enabling individuals to form communities around their experiences of living with AN (Dias 2003). These online spaces and communities vary in terms of the kind of support they offer. One way they are sometimes classified is as either Pro-recovery or Pro-Anorexia (ProAna).

Pro-recovery websites are communities that promote recovery from AN by providing content like recovery testimonials, tips, positive images and slogans, and other kinds of information that supports individuals as they try to move away from AN. In contrast, ProAna websites are seen as enabling AN. They offer a variety of resources for helping individuals develop and maintain practices of disordered eating such as self-starvation: tips, guides, journal entries, motivational content (such as “thinspiration”), and expressions of solidarity and support for individuals trying to maintain an AN way of life. ProAna resources were initially found on dedicated websites, message boards, blogs, and discussion forums. Such ProAna websites tend to be heavily moderated and often intentionally exclusive (e.g., password-protected). However, the ProAna movement has now moved into social media spaces like Instagram and TikTok, where many users share ProAna content in a less hierarchical, more visual, and publicly accessible format (Ging and Garvey 2018, p. 1192).

Since the emergence of ProAna websites, there has been an intense medical and media backlash against such sites (Christodoulou 2012; Davis and Lipsey 2003; Gwizdek et al. 2012; Nagourney 2005). This backlash centers on the idea that ProAna websites do not see AN as a disorder but as a *lifestyle choice*: they provide tips on how to engage in anorectic behaviour more effectively, encourage eating disorders, and fail to recognise the seriousness of AN. Typically, ProAna sites do share tips, promote “Ana” as a lifestyle, and curate “thinspiration content” (e.g., pictures of thin bodies to aspire to). However, this is not the whole picture. Many ProAna websites also include support and encouragement for recovery, as well as provide a broader sense of solidarity, belonging, and understanding to individuals who often feel lonely and misunderstood (Brotsky and Giles 2007; Giles 2006; Norris et al. 2006; Smith et al. 2013). As Firkins et al. (2019, p. 1462) put it: “the assumption that Pro-Ana and pro-recovery websites occupy exclusive polarized perspectives or positions is unhelpful. Indeed, as one would expect, most sites contain pro-illness and pro-recovery elements”. In light of this, while we use the term “ProAna” in this paper, we want to highlight that not all content on ProAna websites or ProAna posts specifically promote anorectic behaviours and practices.

Part of the media backlash and moral panic surrounding the existence of ProAna communities, we argue, stems from a reductive oversimplification of the role they play in individuals’ lives. Much of the coverage of ProAna communities portrays them primarily as *informational resources*. For example, it is common for the content of such sites to be described in the following ways:

So typically what you see on these websites are tips and tricks on how to lose weight, how to hide that weight loss from others, and what’s called thinspiration. (Sharp quoted in Christodoulou 2012).

[ProAna websites] provide individuals with existing eating disorders with information on how to maintain the disorder or even step up their weight loss. (American Addiction Centers website 2020).

As the names indicate, these online communities inform people on how to work anorexic or bulimic practices into their lives. (Scaccia 2018).

In other words, they are seen as places to go and learn how to be a better and more effective anorectic: e.g., to learn techniques and practices for losing weight (calorie counting, rituals surrounding food preparation, etc.), or strategies for hiding the disorder from parents, doctors, and friends (Boero and Pascoe 2012, p. 29). Insofar as they provide information needed to realise an anorectic lifestyle, the thinking goes, these websites bear some medical and moral responsibility for the potential damage this lifestyle can inflict and, therefore, ought to be taken down.

To be clear, these sites do offer this sort of information. As we will see, this is one way they play an important epistemic role in the lives of those who frequent them. However, focusing narrowly on the informational aspect of ProAna sites generates a puzzle: if their primary purpose is to furnish practical information, why do members continue to engage with these communities once they’ve acquired the relevant information (i.e., and become “skilled” anorectics)? In other words, what *else* might these sites offer?

A way to address this puzzle, we propose, is to recognise that ProAna sites provide access to a communal online world with rich epistemic and affective significance. The epistemic significance of such worlds is not simply to *furnish* information (e.g., eating-related tips and strategies) but to reliably *inoculate* members against information that may challenge their anorectic lifestyle. Additionally, these online worlds do not simply scaffold a way of thinking about AN but give rise to a sense of *belonging* and provide emotional support; they provide a place where individuals with AN find “companionship in a safe, anonymous and largely sympathetic environment” (Brotsky and Giles 2007, p. 106).

## ProAna Communities as Epistemic Communities

Rather than adopting the reductive medical assessment of ProAna sites as complex “fact sheets”, we consider these online spaces as providing communities for individuals with AN. While this communal aspect of ProAna sites has been acknowledged by some (e.g. Boero and Pascoe 2012; Brotsky and Giles 2007; Giles 2006), we enrich these discussions by emphasising the *epistemic* and *affective* significance these communities have. We start by exploring the epistemic role these communities can play in the lives of members by drawing on Nguyen’s work on epistemic communities and suggesting that ProAna communities are a form of “echo chamber”.

### Epistemic Bubbles and Echo Chambers

For Nguyen (2020), the categories “epistemic bubbles” and “echo chambers” help clarify how information flows and is often selectively *impeded* in online spaces. An “epistemic bubble”*,* for Nguyen, is a social epistemic structure in which some voices have been excluded (*ibid*., p. 142). Crucially, this exclusion need not reflect ill intentions or questionable motives. It is, rather, an exclusion by *omission*. It results from ordinary processes of social selection and community formation. For instance, as a society, we are increasingly turning to Facebook to get our news. This results in our having inadequate news coverage. Why? Because we tend to be drawn to friends who are similar to us, who share our political or religious views (*ibid*., 143). The more we rely on social networks to filter and present us with news, Nguyen argues, the more we “impose on ourselves a narrowed and self-reinforcing epistemic filter” that excludes views that run contrary to our own (*ibid*., p. 142). We might even be unaware that our network is disconnected from other communities and information sources (Osler and Krueger 2021).

In contrast, “echo chambers” are social epistemic structures where the exclusion of other relevant voices is *intentional*. The inhabitants of an echo chamber share values and beliefs which include reasons to be suspicious of, or reject outright, the voices of those outside the echo chamber (Nguyen 2020, p. 143). Indeed, to even gain membership a “general agreement with some core set of beliefs” (*ibid*., 146) is required. In epistemic bubbles other voices are often not *heard*; within echo chambers, other voices are *excluded—*and, crucially, *actively discredited*. In this way, echo chambers work the way they do by systematically closing off access to outside epistemic sources (Jamieson and Cappella 2008, pp. 163–236). Their mechanisms of exclusion leave members overly dependent on chamber-approved sources of information. The structures of echo chambers are set up to artificially inflate members’ epistemic confidence in these sources and sow and enforce distrust of others. Think, for example, of the way that systematically cultivating a deep distrust of science and expertise is integral to climate crisis deniers and anti-vaccination groups.

Nguyen argues that despite a tendency to conflate them, these two phenomena ought to be kept separate. In part, this is because these phenomena demand different “modes of repair” (Nguyen 2020*,* p. 143). Epistemic bubbles can be popped with relative ease: what is required is exposure to epistemic resources that fill in the informational or argumentative gaps of the bubble.^4^ Echo chambers, on the other hand, are more dangerous, resilient, and significantly more impenetrable. Exposure to new information might dissolve an epistemic bubble. However, Nguyen suggests that encountering dissenting voices is instead “likely to reinforce an echo chamber” (*ibid*., p. 154); for the members of an echo chamber are already primed to expect and discount any disagreement they come across.

Nguyen suggests that one way to escape an echo chamber involves what he calls a “social epistemic reboot” (*ibid*, p. 157). This involves the members of the echo chamber discarding their background beliefs and adopting a new, open, and trusting attitude to testimony coming from sources outside of the echo chamber. As Nguyen notes, this may strike us as both a highly challenging task and something that the member must want or is at least open to. A successful reboot is more likely to take place where the member is not escaping an echo chamber on their own, but instead where an outsider helps the process by gaining the trust of the member and helping motivate a readjustment or reassessment of their epistemic certainty. Nguyen suggests, then, that such a reboot is more likely to be effective when it is a distributed *social* process.

### ProAna Sites

We think that ProAna communities are an example of an echo chamber. First, ProAna sites are not merely repositories for tips on how to engage in “successful” disordered eating practices. They also support and validate AN as a practice or even as a lifestyle choice. For instance, many posts and comments do not simply disavow or deny the potential risks of extremely restrictive eating practices. Rather, they see the end result as worth it—they hold thinness, control, and self-discipline as more important than the associated health risks. Thus, the content of ProAna sites is more intricate than a mere information source, it often promotes a set of epistemic values, beliefs, and practices that its members subscribe to.

Many ProAna sites explicitly state that their community is not for those who simply want to lose weight or look for diet tips. Rather, they are communities for those who *have* AN: “being ana is not for the faint hearted. We will not tolerate noobs, only the most dedicated are welcome here” (PrincessdiANA, quoted in Crowe and Watts 2016). Proving authenticity is an important part of being accepted as a member of many such sites. The term “wannarexic” is applied to members who invoke suspicion for not being “authentically” anorectic and is often levied as an insult—and even used as justification for ousting someone from the community (Boero and Pascoe 2012). One ProAna blog writer, interviewed by Yeshua-Katz and Hård af Segerstad (2020), states that she “doesn’t want to write what could be conceived as “tips and tricks”” by “wannarexics”, as this would invalidate her blog.

On many ProAna sites (as well as in posts shared on social media platforms) this core set of beliefs is encapsulated as a list of commandments labelled the Thin Commandments. These commandments set out the values of the Ana community, emphasising, in particular, the value of exercising control and self-discipline, of being thin, of losing weight (as well as prescribing certain practices such as avoiding eating certain foods, calorie counting, fasting, purging, taking laxatives, and feeling guilty about eating). They are also sometimes supplemented with the Ana Creed, the Ana Psalm, Ana Laws, and prayers or letters to Ana, where “Ana” is portrayed as a protector figure helping her followers live their lives in control (Crowe and Watts 2016; Stapleton et al. 2019). This credal framework highlights the value and belief system that such sites offer and endorse, as well as the monitoring and policing of membership based upon one’s perceived authentic commitment to the beliefs of the community. These qualities fit Nguyen’s description of an echo chamber as demanding a subscription to a set of beliefs in order for membership to be attained.

Moreover, there is significant evidence that other voices are not simply omitted but actively ousted from many such groups, whether these are the voices of so-called “wannarexics” or those critical of ProAna beliefs and practices. Some groups actively work to keep dissenting voices out by making the sites password-protected and involving processes for obtaining and revoking membership (Boero and Pascoe 2012). Indeed, many individuals with AN report joining ProAna communities to escape the pressures or criticism of family and friends who oppose AN practices and beliefs. ProAna sites are described as safe havens, spaces where they can engage with others committed to Ana: “I am tired of everyone telling us that we shouldn’t be like this. I am saying that we are here, we are thin, and that’s ok, it's normal, it's beautiful.” (Bones are my beauty, quoted in Crowe and Watts 2016). One of the allures of such sites is precisely that they do not allow dissenting voices and opinions to be circulated or given epistemic worth.

Importantly, being part of a ProAna community can work to *inoculate* users to the voices of family, friends, and doctors who urge them to eat. Following ProAna rhetoric, it is *because* these “outsiders” do not truly understand what it means to follow Ana that they try to undermine the individual with AN’s self-control and discipline. It is not simply that other voices are not heard. Rather, it is that hearing dissenting voices can work to reinforce an individual’s commitment to Ana. For, in refusing to bow to the pressure to eat, the individual with AN demonstrates their strength, autonomy, and authenticity. Accordingly, dissenting opinions do not burst the echo chamber so much as affirm the idea that individuals with AN are misunderstood and embed members even more firmly within the ProAna community. As such, we can see what Nguyen describes as *disagreement-reinforcement* at play.

Although Nguyen does not stress this point, echo chambers are not only concerning because they create and embed individuals in a fixed and limited epistemic world-view. Our epistemic frameworks impact our actions. So, visiting ProAna sites does not just cement and inoculate epistemic *beliefs* about AN but also helps motivate and sustain disordered eating *practices*. Indeed, ProAna communities often work as active and dynamic scaffolds for such practices, with members offering each other support and encouragement for sticking to the “project” of AN (Osler 2021a).^5^ As one user emphasises: “Being ana is hard. It takes a lot of dedication and life is a daily struggle with food and with people who don’t understand us” (Hunger Hurts, quoted in Crowe and Watts 2016). Users offer encouragement and support for fasting, sympathise when someone is facing pressure to eat from outsiders, share their own stories and experiences in solidarity and as inspiration to others. This is not a static informational space. It is instead a dynamic interactive space that works as an ongoing form of scaffolding for AN values and practices—scaffolded practices that are often reciprocal between members. For example, a recent form of scaffolding that has arisen on ProAna spaces is “gamification” practices on social media platforms such as Instagram. Here, users might make posts pledging to fast for an hour for every like that the post receives, or pledging not to eat any food item that is listed in the comments, or committing to a group fast (Ging and Garvey 2018).

### Therapeutic Implications

Our objective here is not to simply point out that ProAna communities fit the category “echo chamber”. This framework potentially has significance for developing more effective treatment protocols. AN has a particularly high relapse and mortality rate, with successful treatments remaining elusive. Accordingly, it may be helpful to reassess some basic assumptions guiding intervention and treatment strategies (Kuhle 2019, p. 113).

To see how, note first that the APA’s *Practice Guidelines for the Treatment of Patients with Eating Disorders* distinguishes three areas of treatment: nutritional rehabilitation, medication, and psychosocial interventions. While nutritional rehabilitation and medication address nutritional and body weight symptoms, cognitive behavioural therapy (CBT)—currently the most common form of psychosocial therapy for individuals with AN—is designed to address cognitive, behavioural, and affective dimensions of disordered eating (Farrell et al. 2006)*.* CBT therapies presuppose that body image disturbance—i.e., a significantly higher tendency to overestimate body size and weight than healthy controls (Keizer et al. 2013)—is central to AN. Accordingly, they are designed to address core aspects of such a disturbance. These aspects include: size perception (overestimation or exaggeration of overall size, or a particular part or perceived defect); cognitive and affective factors (negative, unrealistic, or disproportionately valued thoughts, beliefs, and emotions about one’s body); and, behavioural factors (avoiding situations that provoke anxiety, monitoring and grooming behaviours, etc.) (Farrell et al. 2006, p. 291). The overall goal of CBT is to identify and gradually change unhelpful thinking patterns that inform these aspects, including faulty core beliefs about body size, thinness, the value of control and discipline, etc. (Kuhle 2019, p. 113).

Given these assumptions, a CBT-based approach might plausibly advocate removing access to ProAna websites insofar as they are echo chambers furnishing information that shapes and sustains these faulty core beliefs. But this may be problematic. If we take Nguyen’s warning about disagreement-reinforcement seriously, we can immediately see a problem with the idea that the best way to handle ProAna sites is to ensure that members’ access to them is immediately revoked. Following Nguyen, simply banning an individual from accessing ProAna sites might have a counterproductive effect. Instead of diminishing or dissolving an individual’s beliefs, it might instead work to: *reinforce* an individual’s epistemic beliefs and commitments; affirm and deepen a sense of not being understood by outsiders; or even be interpreted as a challenge to the individual’s self-discipline and self-control that must be directly addressed and overcome (Firkins et al. 2019). As such, we suggest that a more nuanced approach must be taken when thinking about how to deal with the use of ProAna sites during recovery.

First, what Nguyen’s work suggests in this context is that we should recognise that if an individual is a member of an echo chamber, their epistemic framework is not simply a case of misguided individual beliefs. It is situated in a broader *communal* epistemic structure. As such, addressing “faulty” core beliefs cannot take place in an individualistic vacuum. It must explicitly recognise and address how these beliefs are scaffolded and embedded in that individual’s felt relationship to, and participatory membership within, a broader community—one that confers a sense of trust and solidarity.

Second, Nguyen’s analysis suggests that, in order for the epistemic “rebooting” to be successful, the therapist must be aware that the individual is likely to initially mistrust attempts to make them question their epistemic commitments. Consequently, establishing *trust* will be essential to the therapeutic process. Immediately dismissing the content of ProAna sites, or summarily cutting off all contact with them as the first step toward recovery, may not be the best way to do this. In addition to potentially reinforcing the individual’s epistemic commitments and closing down their openness to voluntarily leaving these communities, such a strategy may also trigger experiences of shame, which may further hinder the therapeutic process (Goss and Allan 2009). Instead, it may be more fruitful—and trust-building—for clinicians to offer supportive, nonjudgmental responses to any disclosures made about ProAna usage (Firkins et al. 2019, p. 1469). By acknowledging the important role that ProAna communities play in the lives of those who frequent them—one that extends far beyond furnishing information—clinicians may recognise that leaving these communities is often a transitional process, one that is not completed until viable alternatives are found. Accordingly, intervention strategies “should include explicit discussions about how to negotiate recovery and identify alternative healthy and supportive relationships” (*ibid*., p. 1470).

## From ProAna Communities to ProAna Worlds

Nguyen’s account of echo chambers helps us build up a more nuanced picture of the epistemic role that ProAna communities play for individuals with AN. However, there is an important feature of these communities that the echo chamber framework does not adequately capture: namely, the affective sense of support, belonging, and comfort that these communities bring to their members. Research on the content found on ProAna sites indicates that “emotional support messages were the most frequent form of social support exchanged in pro-ana blogs” (Tong et al. 2013, p. 417). Many ProAna users cite in interviews that they joined such communities specifically to obtain social and emotional support (Mantella 2007 , p. 28). Even those who initially joined for informational purposes highlight the importance of emotional support as part of their continued membership: “I should add that I used to go to them for tips, but now I mainly go for support and giving support to others in positive ways” (quote from Brotsky and Giles 2007, p. 100). ProAna communities, then, do not only provide a community that shares epistemic beliefs, values, and practices but also provide emotional, social, and affective support and comfort.

Although Nguyen emphasises that echo chambers are *social* epistemic structures, the social and affective dimensions of these structures are not expanded upon in detail. *Why* someone might choose to join an echo chamber is not really considered, especially when one is not already friends with (some of) the members; nor are social or affective motivations considered as factors that might encourage someone to *stay* within such echo chambers. Yet, one does not usually find oneself on a ProAna site by accident (though ProAna content on social media does sometimes show up unsolicited on people’s feeds); they are communities that are specifically sought out. Such sites, in bringing people together who might not otherwise be able to connect easily, open up a shared space for “isolated and marginalized individuals” (Boero and Pascoe 2012, p. 34). The *affective allure* and *affective stickiness* of such spaces need to be recognised if we want to understand how these sites play a role in sustaining AN, and in particular why it might be so difficult to leave such spaces.

We now explore how ProAna communities are not just echo chambers but can be thought of more broadly as online *worlds*. They are worlds in which individuals not only share an epistemic belief system with others but also feel a sense of rootedness, belonging, identity, and interpersonal connection that helps them affirm and sustain AN. We employ here the notions of “world” and “ease” from the work of Lugones (1987, 2003) to add another layer of texture to our analysis of ProAna communities. We suggest that attending to this affective dimension not only provides a better understanding of ProAna worlds but adds an underexplored affective dimension to Nguyen’s work more generally.^6^

### Worlds, World-Travelling and Ease

In her work, Lugones (1987, 2003) discusses the idea that we often do not live in just one world. We routinely negotiate multiple worlds. By “world”, Lugones is not picking out specific geographical locations or destinations. Rather, the worlds Lugones talks about are the *social* and *normative* worlds that we find ourselves in.^7^ We might talk, for instance, about being part of an academic world, a queer world, or the Western or Latino world.

Lugones highlights that we can travel between worlds. She stresses that marginalised individuals, in particular, are very familiar with world-travelling. For instance, she refers to her own experience of being forced to travel between the Latino world of her mother and the Western academic world in which she works. Each of these worlds is structured by different normative expectations, rules, values, and so on. For our purposes, a key point is that when successfully moving between these worlds, an individual undergoes an *epistemic shift*. They are exposed to different worlds of sense as well as gain understanding about different versions of themselves in different worlds (Lugones 2003, pp. 17, 18). Moving between worlds, then, does not just relate to an epistemic shift relative to the norms and values of that world generally. It also involves a more personal epistemic shift in terms of an individual’s self-understanding and identity.

Integrating what we have learnt about echo chambers from Nguyen, we can add that certain worlds might not just involve an epistemic shift when we enter them, but that these worlds might also be structured in ways that not only endorse a particular set of beliefs and values but, additionally, actively exclude and discredit the voices of outsiders. As such, we can characterise some of the worlds Lugones describes as echo chambers. But what, specifically, does Lugones’ notion of “worlds” contribute to this analysis? Lugones explicitly draws attention to the way in which we can feel a greater or lesser “sense of ease” in the worlds that we find ourselves in; we feel more comfortable in some worlds than others. By “ease”, Lugones is referring to instances where we occupy a world smoothly, confidently, and without tension; when we “know the norms…all the words…[and] all the moves” (1987, p. 12), when we are happy to call it “my world” (*ibid.*). As set out below, Lugones suggests various factors that engender a sense of ease in a given world, such as shared language, norm agreement, shared history and human connection with others. When we lack “familiarity, fluency, and comfort in a given world” (Pitts 2019, p. 346), we are at *dis-*ease. The key point is that occupying a world does not just have epistemic significance. It is accompanied by an *affective* dimension.

Lugones (1987, p. 12) stresses that being at ease, while personally comfortable, “tends to be dangerous as it produces people who have no inclination to travel across worlds”. This is because Lugones sees world-travelling, when done in the right way, as a way of opening up one’s experience of self and world possibilities.^8^ In the context of worlds that are also echo chambers, this sense of ease can also be interpreted as yet another barrier to an individual leaving. It is not only the case that a member of such a world distrusts the beliefs and views of outsiders. They also might actively value the comfort, belonging, and sense of identity they experience in such a world—affective factors that diminish their motivation to move to worlds where they feel less at ease.

### ProAna Worlds

As discussed above, ProAna sites do not just offer information; they provide a rich, multidimensional community. These communities have particular norms which the members are expected to adhere to, as well as supporting various practices, beliefs, and commitments. We argue that ProAna communities should be conceived of as worlds. We suggest that entering a ProAna community allows an individual with AN to inhabit a world in which they are comfortable, where they can be open about their eating practices and values, able to share these with others around them, to feel understood and not othered, infantilised, or medicalised. In short, members of ProAna communities are likely to feel *at ease* in these spaces.

Lugones (2003, pp. 90, 91) outlines several factors that may contribute to an individual feeling at ease in a given world. These factors, we argue, are often present for members of ProAna communities. First, Lugones states that one feels more at ease in a world where we share a language with other inhabitants. This does not simply mean that we share a common spoken language (such as Urdu or Portuguese) but also ways of speaking and shared jargon. As already highlighted, ProAna sites are filled with shorthand and jargon that might be confusing for outsiders but familiar to members (e.g. “Ana”, “Mia”, “Wannarexic”). Second, Lugones highlights how ease is experienced when we agree with the norms of a world. Finding others in ProAna communities who subscribe to the same beliefs and values surrounding AN might not only enforce one’s own epistemic commitment to those norms but can make one feel that one has found “one’s people” and feel a sense of comfort and kinship with them.^9^ Third, Lugones emphasises the importance that human bonds have for feeling at ease in a world. We are more likely to feel comfortable in a world where we have friends and companions. When members in ProAna sites form friendships, and not just support systems (Firkins et al. 2019), their sense of community is solidified, further driving a sense of ease in these online worlds. Finally, Lugones notes that having a shared history with others can increase a sense of ease. Such a shared history can be fostered between members online, even though they are not physically face-to-face with one another. This shared history often takes the form of reciprocal self-disclosure. For instance, by sharing common experiences of being attacked by family and friends in relation to their eating practices; hiding certain practices; “failing” a fast, and so on (Tong et al. 2013, p. 419). Commitments to rituals, such as group fasts and living according to the Thin Commandments or the Ana Creed, can also create a shared history between individuals who do not live in the same physical space.

It seems apt to characterise the experience of individuals with AN in their everyday offline worlds as pervaded by dis-ease. A common experience of such individuals is feeling isolated, alone, and cut off from others, as well as being infantilised, medicalised and, sometimes, stereotyped based on their disordered eating (Dias 2003; Malson 1998). This occurs in terms of both *feeling* misunderstood by others but also in practical terms of being cut off from various social practices, particularly those that revolve around food. This feeling of dis-ease can be seen as a motivation for finding a new world not marked by this sense of discomfort.

By attending to these social and affective dimensions of ProAna communities, we argue that it is helpful to think of these ProAna sites not just as providing an epistemic community but also an affectively complex, norm-governed world. The motivation for doing so is two-fold. First, it captures the *richness* of these sites. It marks a distinct contrast between seeing these as (mere) informational sites, versus recognising the important role that these sites can play for those with AN in terms of providing a sense of solidarity, belonging, identity, etc. Second, it helps us understand what we might call the *affective “stickiness”* of these ProAna sites. Individuals do not just experience epistemic confidence and validity by frequenting these sites. They also experience an important sense of belonging and ease in these worlds—an ease that often stands in stark contrast to how they might feel in their offline worlds.

When considering how an individual might be encouraged to stop using ProAna sites, it is important to recognise that we are not only encouraging them to *epistemically* reboot their beliefs and practices surrounding themselves and AN (an already difficult task). Rather, we are encouraging them to leave a world that they feel *at home in*, a world they feel they belong to. Moreover, we are asking them to leave this world of ease in favor of a world that they might experience intense dis-ease in. Feeling at ease in a ProAna world, then, can add an additional component to an individual's reluctance to leave an echo chamber, beyond the epistemic factors Nguyen considers. Individuals, in short, might *like* being part of the world which we want them to leave. Moreover, they might like the self they develop within that world. For instance, an individual with AN might be respected and listened to by other members of a ProAna community; they may be treated with an unaccustomed dignity, as an autonomous and self-determining agent. And this experience, in turn, may stand in stark contrast to the experience of being treated as ill, being infantilised and medicalised in the offline world.

### Therapeutic Implications Revisited

Bringing Lugones into this context can help to develop and refine some of the points about therapy we made previously. Specifically, Lugones’ analysis of world-travelling and ease highlights how establishing trust in a therapeutic context as part of the “epistemic rebooting” in recovery will require more than addressing an individual at the level of their beliefs. As we saw in the previous section, ProAna sites are kinds of worlds—rich, affectively-structured worlds in which individuals with AN may develop a deep sense of ease and belonging. In other words, they are worlds that address individuals' *relational* needs (Firkins et al. 2019). Therapeutic interventions should be tailored accordingly.

A worlds-based perspective can provide some general guidance in this regard. First, this perspective helps further reinforce why conceptualising AN as primarily a cognitive disorder potentially leads to ineffective treatment protocols. CBT approaches (and cognitive approaches more generally), which place faulty beliefs at the heart of disordered eating practices, do not accurately represent the experiential reports of how many individuals live with, and actively sustain, AN as a *shared project* (Kuhle 2019, p. 120). They also overlook the central role online worlds often play in this project. Actually listening and responding to first-person reports of lived practices is, therefore, crucial. As we’ve seen, while cognitive aspects of AN may be important, the narratives of those living with AN often foreground the affective components—e.g., “…I mean I wouldn’t say anorexia is a thought as such. I don’t think I, it’s more of a feeling I, I WANT to do it and I guess in a way it’s almost an EMOTION, anorexia” (Charland et al. 2013, p. 357)—including, crucially, the emotional and social support they receive from ProAna sites (Tong et al. 2013). Intervention strategies, therefore, need to begin with individuals’ experience of AN as their starting point (Kuhle 2019, p. 120).

Second, a worlds-based perspective reminds us that, in coaxing individuals to move away from ProAna sites as part of their recovery process, we are asking individuals to do more than change some of their core beliefs. We are asking them to leave a community they feel a sense of ease and belonging in—a world that meets different aspects of their relational needs. Recovery strategies must therefore involve helping the individual develop and sustain ties to *new* worlds that meet these needs and furnish opportunities for developing a similar sense of ease and belonging.

This observation is crucial because, as Stinson (2019) notes, individuals with AN often “feel misunderstood by their families, friends, and doctors”. One of the women diagnosed with AN interviewed in Malson (1998) claims that “a lot of psychiatrists don’t know what they’re talking about…it shouldn’t be looked at on the surface [i.e., just in terms of beliefs]” (p. 2155). As the previous section made clear, ProAna sites are worlds in which individuals can overcome this sense of isolation by providing opportunities for individuals to develop a shared commitment. While this commitment clearly involves beliefs, it involves other things, too: a feeling that one *belongs*; that one is participating in a legitimate way of life; a sense of identity and purpose; and, an ethos of solidarity and support in the face of critiques from those who don’t understand or approve of these communities. All of these things are part of the general ease individuals feel within these worlds. As such, clinicians ought to familiarise themselves with these communities in order to recognise how they meet individuals’ relational needs and develop ways to emulate some of these world-building strategies within their therapeutic work (Firkins et al. 2019, p. 1469).

## Conclusion

AN is typically treated as an individual disorder, often seen as rooted in an individual's faulty beliefs and/or misperception or misjudgment of their body. In this paper, we have challenged this individualistic perspective. We have sought to shed light on some important ways that other people can support and sustain disordered eating practices in AN. With the rise of the Internet, individuals with AN have unique opportunities not only to become aware of others with AN but also to form online communities with them. These communities, we have argued, do not simply furnish members with AN-related information. They form ProAna *worlds*. These are worlds that are shared with others, worlds that support and sustain epistemic and affective dimensions of AN undertaken as a lived practice. Challenging the cognitivist and individualistic assumptions informing some therapeutic interventions like CBT, we have highlighted the need to consider the key role that others should play in guiding therapeutic intervention and recovery.

While we have explored how interpersonal communities can help uphold and sustain particular values, beliefs, and practices associated with AN, both epistemically and affectively, we want to stress that our account should not be read as suggesting that AN *is* a disorder of faulty thinking or beliefs alone. Elsewhere, we have stressed that AN is a bodily disorder and that understanding how an individual experiences their body is necessary for understanding AN (Krueger and Osler 2020; Osler 2021a). We see our discussion here as complementary to this embodied approach to AN, as providing another layer of texture to our understanding of complex disorder; in particular, as providing a dynamic intersubjective dimension that is often overlooked. To put it another way, in stressing the epistemic and affective role that ProAna worlds play in sustaining AN, we do not want to suggest that AN is, at heart, a cognitive disorder. We want to emphasise that, as embodied subjects, we are always embedded in an interpersonal world, one which works to shape and influence our experience of the world and ourselves. When it comes to understanding AN, then, it is important to consider how an individual’s world(s) can contribute to and sustain a disorder like AN by scaffolding and influencing one’s eating practices, values, identity, and bodily experiences.

Finally, we want to end by emphasising that although we have suggested using Lugones’ work on worlds and ease to complement and enrich Nguyen’s work on echo chambers in the context of ProAna communities, we think that this enrichment extends to the discussion and conception of echo chambers more broadly. While Nguyen provides an insightful and sensitive analysis of echo chambers as social epistemic structures, the affective dimensions of these structures go underexposed. Where Nguyen focuses on how echo chambers work to exclude others through epistemic inoculation against dissenting beliefs, we have drawn from Lugones’ work to underscore the way a sense of inclusion, belonging, and ease can work to attract and embed members within echo chambers and further shore up barriers between members and other worlds. As we have highlighted, echo chambers may be both affectively alluring, in terms of offering someone a community and a sense of belonging, and affectively sticky, for those who find themselves at ease in these worlds. This suggests that leaving an echo chamber might not only involve an e*pistemic rebooting* but an *affective re-worlding* as well. By connecting Nguyen’s and Lugones’ work, therefore, we hope to point the way towards a multidimensional account of echo chambers. Continuing this line of research would involve dedicating more attention to the role of ease, comfort, belonging, loyalty, friendship, and community in establishing and cementing our membership in our social epistemic worlds.

## Footnotes

1 The term “disordered eating” implies that there are well-established “ordered” eating practices. It should be noted that what counts as ordered or disordered will be socially and normatively situated, and that disordered eating is something that happens on a spectrum.

2 For an overview of how material and environmental structures might also play a role in sustaining AN, see Krueger and Osler (2020).

3 Under the diagnostic criteria in DSM-V, an individual can only be diagnosed with AN when self-starvation leads to “significantly low body weight”. There is a notable concern with this approach, as it fails to recognise that someone might be practicing anorectic behaviour before they hit a certain weight threshold and not meet the diagnostic criteria. As Kendall (2020) points out, this is particularly problematic when we consider how AN is typically characterised in relation to white aesthetics regarding body weight, shape, and size.

4 Although we cannot expand on this point here, we suspect that Nguyen is overly optimistic about the ease with which epistemic bubbles can be popped. This is particularly apparent if, say, we take into account the value that an individual might place on being a member of an epistemic bubble in terms of feeling a sense of belonging with others “like you” and the value one might place on certain epistemic positions or practices that relate to one’s sense of self.

5 For a detailed taxonomy of different kinds of scaffolding see: Colombetti (2020), Coninx and Srephan (2021).

6 For an account of *affective* bubbles, inspired by Nguyen’s work, see Roberts (manuscript).

7 For a related discussion of “little worlds” see: von Maur (2021).

8 See Lugones (2003), Ortega (2016), and Sullivan (2004) for a discussion on how world-travelling can be done authentically and inauthentically.

9 For an in-depth discussion of feeling belonging with online communities, see: Osler (2020).
